# Dendritic Cells in the Cross Hair for the Generation of Tailored Vaccines

**DOI:** 10.3389/fimmu.2018.01484

**Published:** 2018-06-27

**Authors:** Laura Gornati, Ivan Zanoni, Francesca Granucci

**Affiliations:** ^1^Department of Biotechnology and Biosciences, University of Milano-Bicocca, Milan, Italy; ^2^Division of Gastroenterology, Harvard Medical School, Boston Children’s Hospital, Boston, MA, United States

**Keywords:** dendritic cells, vaccination, pattern recognition receptors, antigen delivery, adjuvants

## Abstract

Vaccines represent the discovery of utmost importance for global health, due to both prophylactic action to prevent infections and therapeutic intervention in neoplastic diseases. Despite this, current vaccination strategies need to be refined to successfully generate robust protective antigen-specific memory immune responses. To address this issue, one possibility is to exploit the high efficiency of dendritic cells (DCs) as antigen-presenting cells for T cell priming. DCs functional plasticity allows shaping the outcome of immune responses to achieve the required type of immunity. Therefore, the choice of adjuvants to guide and sustain DCs maturation, the design of multifaceted vehicles, and the choice of surface molecules to specifically target DCs represent the key issues currently explored in both preclinical and clinical settings. Here, we review advances in DCs-based vaccination approaches, which exploit direct *in vivo* DCs targeting and activation options. We also discuss the recent findings for efficient antitumor DCs-based vaccinations and combination strategies to reduce the immune tolerance promoted by the tumor microenvironment.

## Introduction

Vaccines represent one of the most effective Copernican revolutions for humankind and world health. This innovative discovery by Edward Jenner in the late years of the XVIII century allowed for control or complete eradication of infectious diseases as smallpox (1979) and rinderpest virus (2011) (1). This immunization strategy posed the bases for current remarkable therapeutic approaches against not only infections but also cancer. In evolutionary terms, pathogens have acquired the capability to circumvent the immune system with several evasion mechanisms, revised elsewhere (2), that prevent pathogen clearance and the establishment of immune memory. Vaccines represent the unique tool we have to impede pathogen spread; therefore, the urgent need for efficient vaccines is as relevant as before. *Mycobacterium tuberculosis*, which causes tuberculosis, is currently one of the most feared infectious agent due to its capability to evade the immune system, leading to death of more than one million of people per year. Unbelievably, the only licensed vaccine against *Mycobacterium tuberculosis* is bacillus Calmette-Guérin (BCG) conceived about 100 years ago. Nonetheless, BCG has displayed some degree of inefficacy in humans, thus raising the need for new tailored vaccination strategies that are currently under investigation (3). Moreover, every year, new cases of human immunodeficiency virus (HIV) infections lead to the necessity of a vaccine to control and prevent the spread of the virus. Up to now, vaccines against HIV have not passed phase II clinical trials due to poor protection conferred, requiring revision of delivered antigens (ags) and strategy to improve T cell response (4). Moreover, the recent outbreaks of Ebola virus and Zika virus infections clearly demonstrate that still nowadays more than few infectious diseases need to be overwhelmed, as reported by the World Health Organization. On the other hand, vaccines represent also a therapeutic tool against cancer. One of the hallmarks of cancer is the capability of tumor cells to evade immune-mediated destruction (5) by promoting a tolerant milieu. Therefore, the immune system has to be pushed to respond specifically and robustly against tumors cells.

To address this purpose, it is becoming more and more evident that dendritic cells (DCs) stand out as a potent tool in our hands, being the mediators of cellular and humoral responses (6). DCs have been discovered in 1973 by R. Steinman and Z. Cohn that divided phagocytic cells (discovered by E. Metchnikoff in 1887) in macrophages and DCs on the basis of different effector functions: microbial scavenging activities for macrophages and antigen-presenting function for DCs (7, 8). Since then, DCs have emerged as the most potent antigen-presenting cells capable of shaping adaptive responses both during infections and cancer. Moreover, the broad spectrum of DCs activation makes them suitable for fine shifting of the type of response the context needs. Taking advantage of new adjuvants, innovative ags-delivery carriers and targeting strategies, it is now feasible to optimize the activation and ag presentation processes by the specific DCs subset that is the most effective in the initiation of the adaptive response needed in a given context. Here, we discuss the diverse phenotypical and functional properties of DCs subtypes that are exploited by recently developed vaccine approaches, dealing with advances in the use of ags, adjuvants, carriers and DCs-expressed molecules, object of targeting.

## DCs Identity: A Multifaceted Functional Family

**Figure 1:**
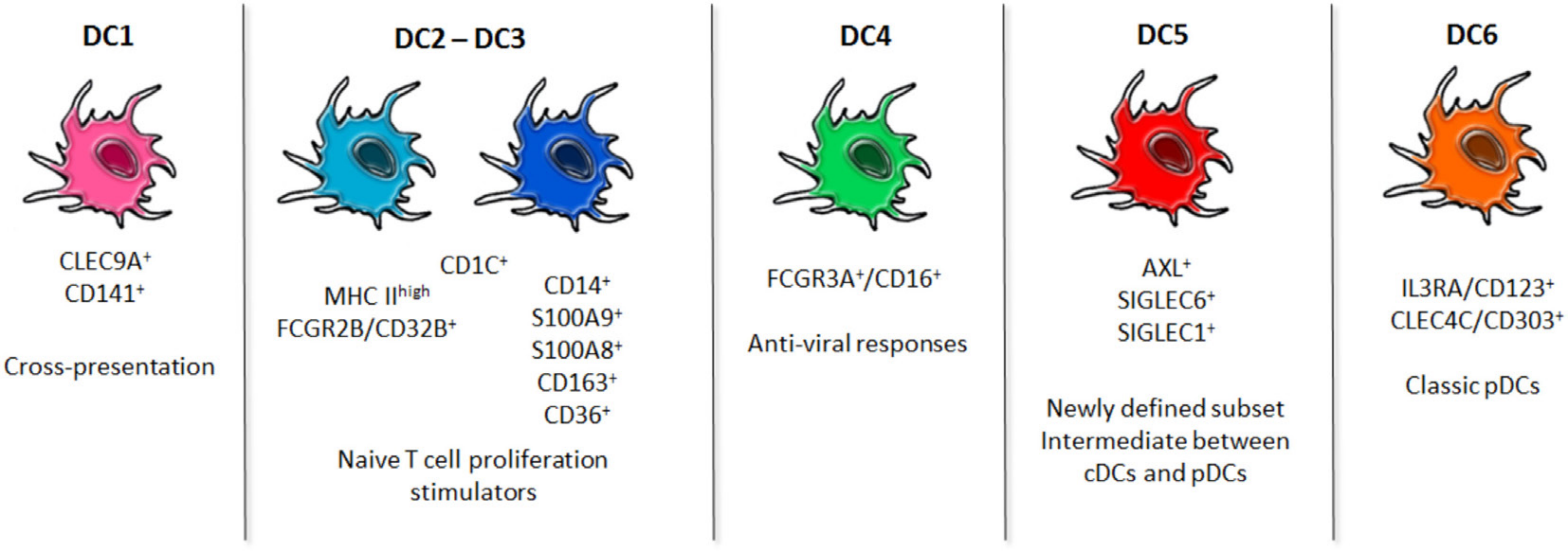
Dendritic cells (DCs) readapted taxonomy. Newly identified populations of blood human DCs are shown. DC1 subset is clearly distinct by the expression of CLEC9A, and it is specialized in cross-presentation of ags. DC2 and DC3 constitute the conventional DCs pool, even though they appear to be phenotypically slightly different and, upon stimulation with TLR ligands, their diversity emerges. DC4 is a population characterized by an upregulated Type I Interferon pathway for antiviral responses. DC5 has emerged as a new population whose specific functions are still unexplored. DC6 corresponds to the classic plasmacytoid DCs. These advances in the fine characterization of DCs in humans may shed light on the best subset to be targeted to incentivize the desired immune response.

Dendritic cells are the primary professional antigen-presenting cells (APCs) that reside in both lymphoid and non-lymphoid organs (9–11). DCs encompass several heterogeneous subsets whose subdivision relies on ontogeny, expression of surface-receptors, and transcription factors (12–14). Much effort has been done in the identification and characterization of tissue-specific DC subsets to unravel the correlation between phenotype, localization, and functional properties, both in health and disease. Initially, DCs have been classified into conventional DCs (cDCs) and plasmacytoid DCs (pDCs). Briefly, cDCs prime naïve T cells and orchestrate ag-specific adaptive responses, while pDCs intervene during viral infections producing type I interferons (IFNs). Advanced approaches have extremely pushed our understanding of DC biology, resulting in a recent readapted taxonomy (12, 15, 16). Indeed, Villani and colleagues identify six subsets of DCs and monocytes in human (Figure 1): DC1 (CLEC9A^+^CD141^+^ DCs), DC2 and DC3 (CD1c^+^ DCs), DC4 (FCGR3A/CD16^+^ DCs), DC5 (AXL^+^SIGLEC6^+^ DCs) and DC6 (pDCs). DC1 represent the cross-presenting CD141^+^/BDCA3^+^ DCs while D2 and D3 correspond to cDCs displaying antigen uptake and processing capabilities. DC4 seem to be more prone to respond to viruses and are phenotypically close to monocytes. DC5 represent a newly defined subset that share features with both pDCs and cDCs, even though they appear to be functionally different from pDCs and more similar to cDCs. Indeed, DC5 localize in T cell zone of tonsils, probably promoting fast adaptive immunity. Due to this fine clustering, DC6 correspond to a more pure pDCs population (12). This precise classification opens the way for a more accurate view of DCs role in pathologies and provides cues for more specific targeting in immunotherapies. Indeed, it is reasonable to assume that this extreme phenotypical diversity correlates with different intrinsic functional properties of DCs, as emerged in Villani’s work (12, 17, 18). In addition, environmental cues dictate DC activation and drive specific T cell responses (19, 20). Indeed, DCs display a plethora of pattern recognition receptors (PRR) that are specifically bound by microbe- or damage-associated molecular pattern (PAMP and DAMP, respectively) (21). Upon receptors engagement in peripheral tissues, the transduction signals lead to DC maturation with the upregulation of co-stimulatory molecules (referred to as “signal 2”) and the pivotal chemokine receptor CCR7 that allows DCs migration through afferent lymphatic vessels to the draining lymph node (LN) (22–24). In parallel, DCs mediate ag proteolysis to present intracellular peptides on major histocompatibility complex (MHC) class I to CD8^+^ T cells and exogenous peptides on MHC II to CD4^+^ T cells (referred as “signal 1”). DCs can present exogenous ags on MHC class I through the so-called cross-presentation, allowing them to induce CD8^+^ cytotoxic T lymphocytes (CTLs) against viruses and tumor cells. Indeed, once in the LN, mature DCs encounter cognate naïve T cells and initiate adaptive responses (25). In the absence of maturation, as in steady-state conditions, the ag presentation and consequent migration to LN promote peripheral tolerance *via* T cell anergy or regulatory T cell formation (26–28). Depending on the receptors engaged, DCs display different maturation states and produce different inflammatory mediators (often referred to as “signal 3”) that impact on the following cellular and humoral responses. The three signals released by DCs drive T helper (Th) cell differentiation. Briefly, DCs educate CD4^+^ T cells against intracellular bacteria by promoting their polarization into IFN-γ-producing Th type 1 (Th1) cells. Upon infection by multicellular parasites, DCs, with the help of basophils, polarize CD4^+^ T cells into Th type 2 (Th2) cells that produce mainly IL-4. For specialized mucosal and skin immunity, DCs drive the activation of Th type 17 (Th17) (29). Thus, polarization of T cells is a crucial event that provides mechanisms specifically orchestrated to restore physiological homeostasis. DCs undergo apoptosis once they have fulfilled their functions. The rapid DC turnover after activation is necessary to avoid excessive T cell activation (30) and to maintain self-tolerance (31, 32). T lymphocyte activation culminates with the establishment of the immunological memory, providing the host with T cells more prone and efficient in responding to a reinfection by the same pathogen or upon tumor relapses (33). Besides, DCs are key players in humoral responses too. Indeed, they directly interact with B cells and indirectly support them by activating CD4^+^ T cells, leading to humoral memory. All these notions strengthen the idea that DCs represent an optimal target for immunotherapies and vaccines, acting at the interface of innate and adaptive immunity.

## Adjuvants Shaping DC Functions

To harness robust responses through DC-targeting vaccinations, DC maturation is essential. Adjuvants become compulsory complement of inactivated or subunit vaccines that may promote suboptimal responses. Furthermore, they improve DC migration, ag availability, and specific targeting. Although it seems clear that immunization could benefit from adjuvant uses, the solely adjuvant licensed in clinics, until recently, was alum (34). Despite alum has been used in vaccination practice since the beginning of the last century, the mechanism through which it activates innate immunity for the subsequent activation of adaptive immune responses remains elusive. The adjuvant properties of alum were initially attributed to the activation of NLRP3 inflammasome (35, 36); nevertheless, further studies have clearly shown the dispensability of NLRP3 and caspase-1 for the generation of responses in the presence of this adjuvant (37, 38). TLR signaling is also dispensable for alum adjuvanticity (39) as well as mast cells, eosinophils, or macrophages (40). Recently, it has been proposed that upon contact with alum, DCs produce IL-2 through the activation of src and Syk kinases, Ca^2+^ mobilization, and NFAT nuclear translocation. IL-2, in turn, is required for optimal T cell priming, activation, and antibody production (41). In addition to alum, other chemical adjuvants have been tested in preclinical models, showing a clear heterogeneity in the responses driven by different adjuvants, independently of the ag (42). This underlies the need of deepening our knowledge on these powerful tools to drive immune responses. Indeed, MF59, an oil-in-water emulsion adjuvant, that allows long-lasting ag retention in draining LN and enhanced ag uptake by LN-resident DCs, promotes robust humoral responses *via* follicular DC activation (43) and CD4^+^ T cell immunity induction (44). Conversely, IC31 adjuvant, which consists of an antibacterial peptide and a synthetic oligodeoxynucleotide (ODN), elicits IFN-β release by human DCs *via* engagement of endosomal TLRs supporting immunity against intracellular pathogens and cancer (45).

In the last decades, attention has been focused on TLR ligands as adjuvants. Currently, several compounds are under investigation: Pam2CSK4, Pam3CSK4, or analogs as TLR2/6 or TLR2/1 ligands (46, 47), Poly(I:C) and similar compounds acting on TLR3 (48, 49), TLR4 agonists (50), Flagellin acting on TLR5 (51), Imiquimod and other TLR7 ligands (52, 53), TLR8 agonists (54), CpG ODN binding TLR9 (55, 56). Due to the possible reactogenicity that may be induced by administering TLR agonists, some compounds are chemically modified to reduce toxicity or are delivered specifically to the DC subsets of interest, avoiding TLR ligand dissemination. Monophosphoryl lipid A, a low-toxicity molecule derived from lipopolysaccharide (LPS), displays promising effects for vaccine design (57) even though it promotes terminal differentiation of CD8^+^ cells, leading to reduced memory protection (58). Another LPS-analog is 7-acyl lipid A that has emerged as potent inducer of IFN-γ-mediated ag-specific responses when co-delivered with poorly immunogenic tumor ags (59).

To improve the effectiveness and strength of immunity, in addition to the efficiency of APC, activation and ag processing and presentation of other aspects should be taken into account. The importance of DC-derived IL-2 in the activation of adaptive responses has been shown not only in alum-driven immune responses and in mouse models of infections (60, 61) but also in tests of human T cell priming in the presence of activatory DCs. During the first few hours after interaction with T cells, activatory monocyte-derived DCs (MoDCs stimulated with the cytokine cocktail, TNF-α, IL-6, IL-1β, and PGE_2_) produce IL-2 and CD25 (62). DC-derived IL-2 is, in turn, trans-presented to T cells at the immunological synapse *via* CD25. Since naïve T cells start to express CD25 only many hours after ag encounter, the DC-mediated presentation of the IL-2/CD25 complex is indispensable for an efficient T cell priming (62). It has been proposed that this is the reason why approved therapies based on the use of anti-CD25 antibodies to avoid the acute phases of autoimmune diseases, or acute rejection of kidney, heart, and hand transplants, are so efficient in interfering with T cell priming or T cell reactivation (62). Since IL-2 is produced in NFAT-dependent manner to improve the adjuvanticity of PRR agonists for vaccination purposes, the capacity of selected PRR agonists to induce NFAT signaling pathway activation and IL-2 production should be considered. Many PRR ligands have been shown to activate the NFAT transcription factor family members in innate immune cells (63). The NFAT pathway is activated in neutrophils, macrophages, and DCs in response to curdlan (64, 65), it is also activated in DCs in response to LPS (30) downstream of CD14, it is activated in response to mannose-capped lipoarabinomannan (Man-LAM), a major lipoglican of *Mycobacterium tuberculosis* (66), and downstream of TLR9 in response to β-glucan bearing fungi (67). The production of IL-2 by innate immune cells during inflammatory responses is relevant not only for an efficient T cell priming but also for the skewing of T cell activation toward type I responses. In mice, DC-derived IL-2 is one of the cytokines required to elicit IFN-γ production from NK cells both in LPS-mediated inflammatory conditions and during fungal infections (68–70). IFN-γ potently activates macrophages and favors Th1 commitment of CD4^+^ T cells. Therefore, early IFN-γ release by NK cells is not only crucial for controlling a variety of primary bacterial and fungal infections but also for the induction of type I immunity and memory, fundamental for the protection against bacterial, fungal, and viral infections and in antitumor immune therapies.

Another important reason for considering the capacity to activate the NFAT pathway in adjuvant selection tests is represented by the fact that NFATs regulate also the production of the prostanoid PGE_2_ by activated DCs (71). PGE_2_ promotes activated DC migration (72) and sustains vasodilation and local edema formation during the inflammatory process. This is particularly relevant for vaccination purposes since the increase of the interstitial pressure generated by the edema forces the fluids into the afferent lymphatics and favors a first wave of antigen arrival to the draining LN (71). Intriguingly, LN drainage of proteins or antigens occurs very rapidly after subcutaneous, intradermal, and intramuscular immunization (73–75), thus permitting an extremely fast uptake by phagocytes strategically localized in close proximity to the subcapsular sinus or lymphatic sinus of draining LN (76–79).

Antigen-presenting cells in LN then maintain the homeostasis of LN themselves and activate adaptive immune responses. In the last decades, the long-held paradigm of migratory DCs, resident in peripheral tissues as the skin, as unique APCs involved in T cell immunity has dramatically changed. Indeed, CD169^+^ subcapsular sinus macrophages, medullary macrophages, and LN-resident DCs are LN sentinels that avoid excessive pathogen dissemination (80, 81) and mediators of immune responses (76).

Concerning migratory DCs and considering the skin, which represents the site of utmost importance for vaccination strategies due to the ease accessibility and the extremely high presence of DCs, skin-resident DCs have been subdivided into epidermal-resident Langerhans cells (LCs), which are Langerin^+^ and two diverse subsets of dermal (d)DCs: Langerin^+^ CD103^+^ and Langerin^−^ CD103^−^ (14, 82). Upon infection, dDCs migrate to the LN within 10–24 h while LCs within 48–72 h, supporting long-lasting ag-presentation. Several works reveal the intrinsic differences between the two subsets in inducing Th or CTL responses, due to the particular cross-presenting capabilities of CD103^+^ dDCs, for instance (19, 20, 83). Once in the LN, whose strategical architecture enhances the probability of encounter between migratory DCs and cognate naïve T cell, adaptive immunity is initiated. Of note, LN-resident DCs are sufficient to promote early adaptive responses independently of migratory DCs when pathogens or antigens directly access the lymphatic conduits (76, 84, 85). In antiviral responses, CD8α^+^ LN-resident DCs play a crucial role, thanks to their intrinsic capability of cross-presentation to CD8^+^ CTL (86, 87) that may be supported by pDCs (88). In Herpes Simplex Virus (HSV) skin infection, CD8α^+^ LN-resident DCs uptake cargo-antigens, ferried by skin-resident migratory DCs in order to elicit CTL (89). Indeed, LCs and dDCs synergize with CD8α^+^ LN-resident DCs, which stand out as the most potent CTL inducers, preferentially sustaining CD4^+^ Th responses both in influenza (90) and HSV cutaneous infections (91). In addition to CD8α^+^ LN-resident DCs, CD103^+^ dDCs display intrinsic capability of cross-presentation, as their human counterpart, CLEC9A^+^ CD141^+^ DCs (92–96). Besides, some authors demonstrated that blocking DC migration from the skin hinders CD4^+^ T cell activation in response to subcutaneous bacterial (97) or soluble antigen challenge (98). Ablating Langerin^+^ dDCs reduced T cell immunity strength, corroborating the notion that migratory DC complement LN-resident DC effects on adaptive responses (99–101). Nonetheless, the roles of LCs in activating T cells are still uncertain, probably due to the controversial functional properties of this innate subset (102–104). Despite this, the synergic effects of LN resident and migratory DCs seem to be undoubted (25, 105). Indeed, Allenspach and colleagues reported that ag presentation by LN-resident DCs few hours after the infection is required to entrap ag-specific T cells in the draining LN and to favor an optimal activation of T cells by migratory DCs that arrive at the LN many hours later (106).

It emerges, therefore, that another aspect to be considered for the identification of efficacious adjuvants concerns the type of DC subset to be targeted and the consequential effects that adjuvants imprint on that subset. Adjuvants play a pivotal role in determining tissue-resident DC mobility to draining LN and efficiency of T cell polarization. Indeed, dDCs acquire mobility after subcutaneous injection of Th1-specific adjuvants as CpG and LPS, but not with Th2-specific ones, as papain, or following contact sensitization with dibutyl phthalate and acetone. Moreover, dDCs are sufficient to promote Th1 and Th2 responses, while LCs are only supportive of Th1 (107). This evidence underscore that, in addition to the polarizing capabilities of adjuvants, also the targeted DC subset must be considered to elicit specific adaptive immunity. Indeed, Antonialli and colleagues reported differential immune responses when CD8α^+^ and CD8α^−^ DCs were targeted with the same ag and adjuvant, either CpG ODN or Flagellin (108).

In addition, to enhance the efficacy of vaccination, the coincident delivery of ag and PRR adjuvants to APCs plays a crucial role. Encouraging evidence highlights the importance of conjugation of ag with PRR adjuvants, since it improves ag uptake, humoral and cellular responses when compared to vaccination with ag co-delivered with free TLR ligands (109). These findings strengthen the notion that adjuvants are formidable chiefs in shaping immune responses and must be selected for the outcomes they promote, in chemical association to the ag of interest.

## Novel Strategies of Vaccination: Multitasking Carriers

**Figure 2:**
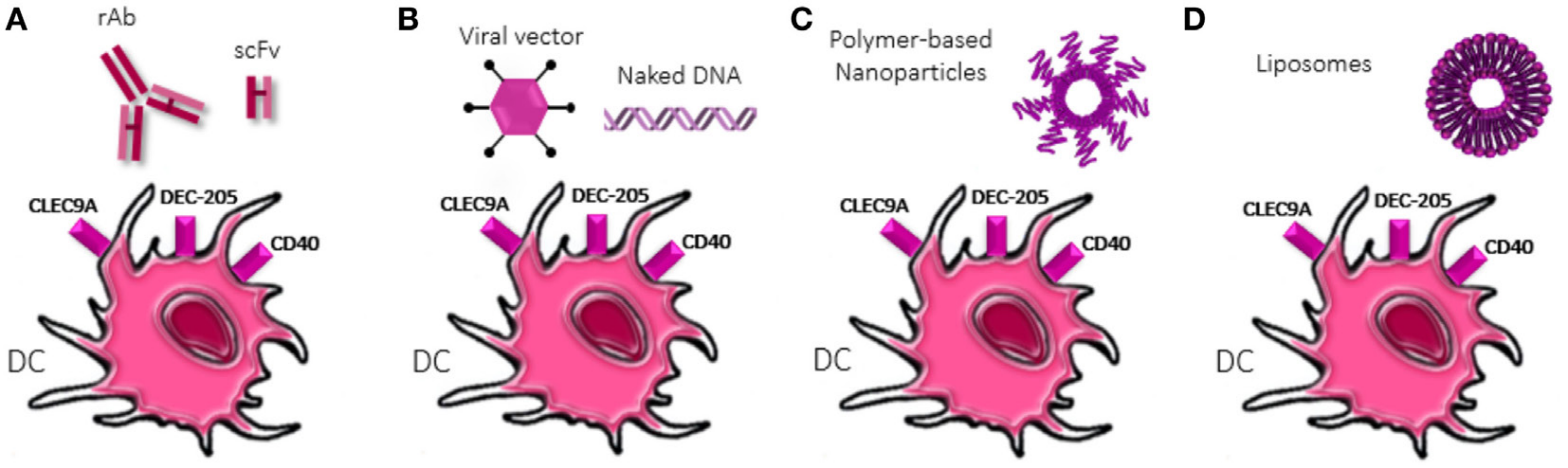
Strategies of dendritic cells (DCs) targeting. Diverse approaches to deliver antigens to DCs are shown. **(A)** Recombinant antibody or single-chain variable fragment (scFv) specific for DC receptors are chemically conjugated with antigen and adjuvant molecules. scFv reduced dimension confers them higher tissue-penetrating properties. **(B)** Viral vector-based vaccines or naked DNA exploit the encoding machinery of DCs to translate antigens, adjuvants but also co-stimulatory molecules (“signal 2”) and cytokines (“signal 3”) increasing the activatory profile of DCs. Naked DNA could be delivered conjugated to nanoparticles (NPs) and liposomes. **(C)** Polymer-based NPs display physical and chemical properties that allow encapsulation or conjugation of antigens and adjuvants as well as ligands for specific DC receptors. Different polymer compositions provide diverse properties and dimension, allowing easy diffusion and/or retention in lymph node. **(D)** Liposomes allow both the encapsulation and intercalation in the phospholipid bilayer of antigens and adjuvants, depending on their chemical properties, as well as the functionalization of the surface with ligands of DC receptors.

The traditional vaccination approaches consisted in the administration of live or attenuated micro-organisms. Up to now, several innovative strategies have emerged to address the need for efficient vaccines, especially against diseases that are critical to treat, as cancer and the infectious diseases already mentioned. The main purpose is to convey ag, adjuvant, and targeting-molecule in a unique compound to increase the efficacy of the ag-specific immune response. To address this issue, different approaches have been explored or are currently under investigation, as shown in Figure 2.

Recombinant antibody (rAb) represents a feasible option. This approach exploits the possibility to chemically fuse peptide ag, adjuvant, and targeting-molecule to Ab to tailor DCs targeting (110–112). In addition to rAb, single-chain fragments variable (scFv) revealed to be an appealing strategy due to their reduced size and enhanced infiltration into tissues, as in solid tumors (113).

Other approaches involve the use of nano-carries as vehicles. The most promising solution to target phagocytes is indeed the use of particulate materials (114, 115). Nanoparticles (NPs) are the best candidates as delivery system, since they can be manipulated to efficiently and predominantly target phagocytes. This is possible, thanks to the versatility of NPs due to: (i) the large amounts of existing different nanomaterials; (ii) the possibility to adjust their size, morphology, and deformability with great precision; (iii) the possibility to load virtually any different type of drug molecules (116).

Viral vectors-based vaccines or virus-like particles rely on the intrinsic capability of viruses to infect cells and exploit their protein-encoding machinery, allowing expression in the cytosol of the engineered plasmid-genes, as ag, costimulatory molecules, cytokines, and adjuvants, providing the bases for strong CTL induction (117). On the other hand, naked DNA can be directly injected or conjugated to nano-carriers to favor specific targeting. The easy designing of nano-carriers-based vaccines along with their multi-component loading feature improve targeting of specific subsets (118) and shape immune responses (119, 120), favoring their application in several fields. In a cancer setting, nano-carriers allow to avoid killing of healthy cells, by delivering tumor ags or DNA encoding these peptides to APCs, inducing specific antitumor responses. Indeed, NPs allow endocytosis and MHC presentation on both class I and II (121) eliciting broad adaptive immunity, even against cancer cells. Rosalia and colleagues designed a polymer-based biodegradable poly(lactic-co-glycolic acid) PLGA NPs loaded with ag, Pam3CSK4, and Poly(I:C) and coated with an agonistic αCD40-monoclonal Ab (NP-CD40). This multi-functional strategy resulted in efficient and selective delivery of NPs to DCs *in vivo* upon s.c. injection and induced priming of CD8^+^ T cells against tumor associated ags, increasing tumor-bearing mice survival (109). PLGA NPs carrying the poorly immunogenic melanoma-derived antigen tyrosinase-related protein 2 along with 7-acyl lipid A, manage in breaking the immunotolerance acting against tumor-antigens. Indeed, administration of the abovementioned NPs resulted in antigen-specific CD8^+^ CTL responses, characterized by IFN-γ production and increase of pro-inflammatory cytokines in the tumor microenvironment (TME) (59). Another nano-carrier-based approach relies on liposome, self-assembled vesicles composed by lipid bilayers with high functionalizing properties. Besides, Maji and colleagues reported that after uptake by DCs, cationic liposomes localize in endosomal compartments that allow ag presentation preferentially on MHC I but do not exclude MHC II ag presentation (122), suggesting a crucial role in antitumor or antiviral immunity supported by Th responses.

In addition to the use of NPs, targeting DC-specific receptors has become an attractive strategy for vaccine development due to the enforced efficiency of immune responses when compared to generic-delivering approaches. Here, we report the more characterized DCs receptors, currently under investigation in the scenario of tailored-vaccination, as shown in Table 1.

**Table 1:** Targeted receptors for tailored ags delivery.

	Receptor	Expression	Activity	Clinical trials
CLEC9A	C-type lectin receptor	Human: CD11c^+^ CD141^+^ XCR1^+^ conventional DCs (cDCs) CD14^+^ CD16^-^ monocytes MOUSE: plasmacytoid DCs (pDCs) XCR1^+^ CD8α^+^ lymph node-resident dendritic cells (DCs)	Major histocompatibility complex (MHC) class I MHC class II Ag presentation	–
DEC-205	Endocytic receptor	Human: cDCs, monocytes, B cells MOUSE: CD8α^+^ DCs Dermal/interstitial DCs Langerhans cells	MHC class I MHC class II Ag presentation	NCT03358719: recruiting NCT01834248: completed NCT02166905: recruiting NCT01522820: completed
CD40	Transmembrane glycoprotein Surface receptor	Human/mouse: cDCs and pDCs, monocytes, B cells, endothelial cells	DCs activation	NCT03329950: recruiting^a^ NCT02706353: recruiting NCT03214250: recruiting NCT03389802: recruiting NCT03418480: recruiting NCT03123783: recruiting

*^a^There are currently more than 30 clinical trials involving the anti-CD40 antibody. Here, the more recent trials regarding DCs-based vaccination strategies are reported*.

CLEC9A or DNGR1 is a C-type lectin receptor that mediates endocytosis, but not phagocytosis, with low pH endosomes promoting the drift toward cross-presentation. Importantly, CLEC9A binding of antigens induces antigen presentation on both MHC I (cross-presentation) and MHC II. It is highly and specifically expressed on CD11c^+^CD141^+^XCR1^+^ cDCs and CD14^+^CD16^−^ monocytes in human and in murine pDCs and XCR1^+^ CD8a^+^ LN resident but not CD103^+^XCR1^+^ migrating DCs (123, 124). Indeed, CD141^+^XCR1^+^ DCs constitute the human counterpart of CD8α^+^ XCR1^+^ murine DCs (125). They share XCR1, the receptor of XCL1. XCL1 is released by activated T cells and the axis XCR1–XCL1 is necessary for robust CTL responses (126). CD141^+^XCR1^+^ DCs are the main cross-presenting DCs in human, thus they appear promising for CTL-mediated responses, in tumors and viral infections (127). This specific subset is characterized by the expression of TLR3 that may be exploited to fully activate CLEC9A^+^XCR1^+^ DCs since antibody binding of CLEC9A leads to its rapid internalization but not TLR-pathway activation, preventing pro-inflammatory cytokine production and full maturation of DCs (127). Conversely, Caminschi and Li independently demonstrated the potentiality of targeting Clec9A that resulted in enhanced humoral immunity independently of TRIF-MyD88 or TLR4 pathway, even in the absence of adjuvants (128, 129). Targeting Clec9A induces enhanced CD4^+^ T cell proliferation *in vivo*, which supports B cell immunity, when compared to the targeting of another endocytic receptor, discussed later, DEC-205, independently of the use of adjuvants as CpG (130). Some years later, different authors demonstrated that this strong humoral response is endorsed by the establishment of follicular T helper cells memory, even upon vaccination with glycoprotein D of HSV, both in mice and non-human primates (128, 131, 132). These promising results were confirmed also in a human *in vitro* setting, on CD141^+^ DCs (133). Finally, the efficacy of targeting Clec9A has been evaluated in the delivery of poorly immunogenic virus-derived antigens. Park and colleagues managed in conferring specific humoral response, protective upon reinfection (134). Thus, exploiting the specific expression of this receptor on the most specialized DCs in cross-presentation in combination with TLR3 ligands, will enhance antiviral and anticancer responses (135), combined with robust humoral immunity.

DEC-205 or CD205 is a 205 kDa endocytic receptor that has a cysteine-rich domain, a fibronectin type II domain, and 10 C-type lectin-like domains, as well as an internalization sequence in its cytoplasmic tail (136). Thus, it mediates cross-presentation through clathrin- and dynamin-dependent receptor-mediated endocytosis. Indeed, it is expressed by the most professional cross-presenting DCs, the CD8α^+^ DCs subtype, while CD8α^−^ DCs display very low level of this receptor. In addition, DEC-205 is found on dermal/interstitial DCs and LCs (137), thus guaranteeing ag delivery to both skin-resident and LN-resident professional APCs. In humans, DEC-205 is shared among cDCs, monocytes, and B cells, while pDCs, granulocytes, NK cells, and T lymphocytes express low levels of this receptor (138). In addition, DEC-205 regulates molecule recycling through late endosomes, promoting also MHC II presentation to CD4^+^ T cells in LCs (139). Steinman and Nussenzweig have addressed this molecule to improve vaccine efficacy since 2000 (140). By taking advantage of anti-DEC-205 rAb conjugated to OVA peptide, they demonstrated that s.c. injections of this compound lead to a strong IFN-γ and IL-2-mediated immunity only when DCs activation was supported by αCD40 mAb, otherwise, tolerance against the OVA peptide occurs (141). Indeed, diversely from PRR agonists, antibody crosslinking the DEC-205 does not induce DCs maturation (142). Furthermore, few years later, the combined strategy of anti-DEC-205 and αCD40 was reported to confer protection against melanoma and intranasal influenza infection (112). In a viral setting, anti-DEC-205 rAb chemically coupled with HIV p24 gag protein tested *in vitro* on blood cells derived by 11 HIV-infected donors has revealed efficient expansion of IFN-γ-producing CD8^+^ T lymphocytes (143) from all the different donors. This indicated that DCs and CD205 can lead to the generation of different peptides from a single protein. Moreover, vaccines based on the filamentous bacteriophage fd presenting an αDEC-205 scFv, efficiently induce DCs maturation *via* the activation of the TLR9-MyD88 pathway (144), without adjuvants and further elicit potent antitumor responses when compared to non-tailored ag delivery (145). Intriguingly, DEC-205, orphan of a specific ligand, has been proven to be necessary for CpG uptake and eventual DC activation (146).

CD40 is a molecule belonging to the TNF receptor family, expressed by several cell types and among these, DCs. It has emerged as a receptor for the human chaperone Heat shock protein (Hsp) 70 that mediates the internalization of peptides bound to Hsp70 itself (147). Moreover, upon activation, T cell transiently expresses CD40L allowing cross-linking of CD40 on DCs and completing their maturation. From these notions, CD40 appeared an interesting molecule to target for DC-based vaccination strategies. Indeed, by engineering antibody chemical structure, Schjetne and colleagues demonstrated the efficacy of CD40 engagement conferring protection against myeloma- and lymphoma-derived ags (148). Moreover, through the co-administration of two DNA-based vaccines encoding either CD40 and the foot-and-mouth disease-derived ags, the transient increase of endogenous αCD40 antibodies allows an efficacious DCs activation and an efficient development of ag-specific T cell immunity, if compared to the administration of DNA encoding ags alone (149). Further promising results have been obtained in a vaccine against cyclin-D1 that is overexpressed by mantle cell lymphoma (MCL). Thanks to algorithm analysis, Chen and colleagues identified three cyclin-D1-derived peptides that efficiently bind to MHC class I of DCs, potentially overexpressed in all MCL patients. By generating a rAb targeting CD40, they efficiently delivered these tumor associated ags to DCs and mounted IFN-γ-specific T cell responses in patients-derived peripheral blood mononuclear cells (150). Thus, CD40 represents a specific DC-targeting molecule that has been used in combination with other targeting approaches to support specific DCs activation, avoid tolerance, and induce robust T cell immunity (110, 141).

## DCs and Cancer

When evaluating vaccination strategies for cancer patients, it is compulsory to take into account one of the hallmarks of cancer: avoiding immune destruction by promoting tolerance and disarming the immune system (5). The orchestration of antitumor responses involves multiple protagonists and mediators, among these, cytotoxic T cells and NK cells, whose activation is supported by DCs (151). Furthermore, DCs-based vaccines has emerged as more efficient in promoting T cell immunity if compared to peptide-based vaccination approaches (152). Thus, much effort has been made to improve strategies of DCs-based vaccination in neoplastic diseases, to ameliorate the prognosis or eradicate both primary tumor and metastases. Up to now, two different approaches have been addressed: *ex vivo* generation of autologous pulsed DCs and direct *in vivo* targeting of DCs, as previously discussed. The former strategy provides a better control of the maturation and activation state of DCs and a specific load of the ag to the selected DCs subset. Despite this, intense work is needed to generate this vaccine, since it is personalized for each patient and only few subsets of DCs are feasibly generated *in vitro* or collected *ex vivo*, limiting the access of ags to other more functionally driven subsets. Diversely, the *in vivo* targeting methods allow the generation of large amount of vaccine in a one-step procedure, and the targeting of diverse DCs subsets in their natural environment.

Once the DCs-based vaccine is generated, the efficacy of antitumoral responses has to be evaluated. It is mainly related to (i) the capability to establish specific antitumor-associated ag (TAA) immunity and (ii) the overcome of the tolerogenic status promoted by the TME.

To select highly immunogenic ags, multiple solutions have been tested: whole tumor lysate or killed tumor cells, synthetic long peptides (SLPs), full length proteins, transfection or electroporation with DNA or mRNA coding for TAA, transduction with lentiviral vectors and neoantigens. The availability of an elevated number of antigens through the incubation of DCs with whole tumor lysates or autologous tumor cells allows the presentation of multiple epitopes, loaded on both MHC class I or II, which leads to Th and cytotoxic responses. Indeed, several clinical trials are currently evaluating the benefits obtained by using this approach (NCT01875653; NCT00045968; NCT02496520). SLPs are 28–35 aa long peptides cross-presented by DCs (153), currently under investigation in both preclinical and clinical setting. Compared to short synthetic peptides, the use of SLPs lacks the necessity to know the patients’ HLA haplotype, thus permitting their full exploitation in a larger cohort of people. Moreover, SLPs administration to DCs leads to an enhanced CD8^+^ T cells activation since, once engulfed, they rapidly escape from the endolysosome to follow the path of MHC class I presentation, fundamental in antitumor responses. Indeed, SLPs and DCs-based vaccines are showing promising results in terms of safety and immunogenicity, in both preclinical and clinical settings (154). They have gained attention in the context of human papilloma virus cervical (155), ovarian (156), and colorectal cancer (157, 158), displaying immunogenic capacities, in terms of antibody production and CD4^+^ and CD8^+^ T cell activation, when delivered with adjuvants, as poly ICLC, Montanide-ISA-51 (NCT02334735), and IFNα. When comparing SLPs and full length proteins, it has emerged that DCs process SLPs better that full length protein, due to the slower processing route the latter display (154). Concerning transfection or electroporation of DCs with mRNA or DNA encoding, not only for TAA but also for costimulatory molecules and cytokines, to enforce adaptive immunity, has proven to be efficacious in inducing antitumor CD4^+^ and CD8^+^ T cells expansion, mediated by DCs targeting (159). A similar approach regards *in vivo* lentiviral transduction of DCs, which displays versatility for gene delivery and efficient transduction for non-dividing cells, as DCs. Indeed, Bryson et al. conceived a multifunctional vaccine composed by a modified lentivirus, whose glycoproteins can directly target DC-SIGN on DCs, loaded with breast cancer ags, alpha lactalbumin, and erb-b2 receptor tyrosine kinase 2. Single injections of the compound provided tumor self-ags-specific CD8^+^ T cell immunity, reducing tumor growth (160). Despite the improvements derived by these advanced strategies, in the last years, neoantigens are becoming more and more appealing (161). During tumor progression, cancer cells give rise to neoantigens, novel ags different from the self-tumor ags, derived by the tumor-specific mutations. Therefore, prediction tools, RNA mutanome, and deep-sequencing have allowed the identification of specific non-self-ags that are fundamental in strong T cell immunity (162–164). Indeed, several clinical trials are currently investigating the potential of neoantigens (NCT0235956; NCT01970358; NCT02149225; NCT02348320; NCT02316457). As emerged, different strategies of ags selection have been explored and, even though one strategy may result in a more enforced antitumor immunity if compared to another, still the issue of the TME negative influence on the immune system has to be faced. Indeed, the TME actively suppresses the activation of the immune system. Tumor cells secrete immunosuppressive cytokines, as vascular endothelial growth factor (165, 166), macrophage colony-stimulating factor (167), transforming growth factor β (TGF-β) (168), and IL-10 (169, 170). Even though some of these cytokine display controversial roles, depending on the pathological context, they generally promote DCs tolerogenicity, by limiting their activation and increasing their expression of pro-tumor molecules, such as programmed cell death 1 (PD-1) and indoleamine 2,3-dioxygenase (IDO). Therefore, tolerogenic DCs lead to T cells anergy, Tregs expansion, and Th1 responses inhibition. Phenotypical characterization of immune cells isolated from breast cancer patients, highlighted the functional alteration in DCs, T, and NK cells in promoting antitumor responses (171). Furthermore, tumor cells retain DCs into the TME, preventing their migration to draining LNs and promoting metastatization (172). To address this issue, some *ex vivo* generated DCs-based vaccines are directly administered intranodally, as for the CD1c^+^ DCs pulsed with HLA-A2.1-restricted tumor peptides administered to patients with stage IV melanoma (NCT01690377), which generated tumor-specific CD8^+^ T cells responses and further improvement of survival (173). To reduce the tolerogenic influence of the TME on DCs, the positive role of GM-CSF in improving DCs survival and responsiveness is currently exploited in some clinical trials like a phase I/II trial with a DC/tumor cell fusion vaccine administered in association with GM-CSF to treat renal cancer (NCT00458536). Similarly, others are focusing their attention on FMS-like tyrosine kinase 3-ligand (FLT3L), another crucial DCs growth factor, in combination with other compounds (NCT01811992; NCT01976585; NCT02129075; NCT02839265). FLT3L has, indeed, been shown to increase the efficacy of proteins- and RNA-based vaccines, due to a maturation effect on DCs (174–176). Additional efforts made to counteract the tolerogenic influence of the TME include the use of PD-1 and IDO inhibitors. Co-administration of anti-PD-1 molecules increases the efficacy of DCs-based vaccines, in terms of enforced intratumoral CD8^+^ T cell responses and trafficking of CD8^+^ memory T cells, as observed in a preclinical model of glioblastoma (177). In parallel, several clinical trials are aiming at evaluating the efficacy of DCs-based vaccines combined with anti-PD-1 agents (NCT03014804; NCT03325101; NCT03035331). The other tolerogenic marker addressed in cancer immunotherapy and DCs-based vaccine is IDO. Indeed, silencing approaches to reduce the expression of IDO in DCs for vaccination in preclinical models, have resulted in decreased T cell apoptosis, reduced numbers of Tregs, decreased tumor size when compared to mice that had received ags-loaded DCs without IDO silencing (178). IDO inhibitors in DCs vaccination are currently being tested in phase II clinical trials (NCT01560923; NCT01042535).

All these approaches have explored different scenarios to evaluate the more efficient therapeutic combination that seems to move toward personalized vaccinations for cancer patients.

## Concluding Remarks

In this review, we have underscored the crucial role of DCs in orchestrating immune responses and; therefore, the great interest in targeting these cells in novel vaccination strategies. We have reported examples of different approaches aimed at amplifying the efficiency of immunizations against cancer or infectious diseases. Indeed, the urgent need of vaccines is as relevant as before because of newly emerging diseases with ineffective current therapies. Deepen the mechanisms underlying these pathologies may provide cues on the more appropriate design of vaccines and by merging diverse tailoring strategies we could enforce the immune system. As a matter of fact, it is suggested to act on different fronts when designing new vaccines, since several factors must be considered: (i) targeting DC subsets specialized in initiating the desired cellular or humoral immunity/memory; (ii) adjuvants that strengthen and drive T and B cell responses; (iii) fine and optimized selection of the immunogenic ags to drive enforced responses; (iv) novel strategies to convey ags and adjuvants to DCs; (v) route of administration. Starting from these notions, in the last decades, enormous efforts have been made to tailor vaccination strategies. New technologies as well as recent advances have allowed extreme flexibility in designing vaccines and shaping the following outcomes. Nowadays, researchers do have smart tools to manipulate immune responses with prophylactic or therapeutic vaccinations. The abovementioned findings pave the way for possible therapeutic approaches, theoretically applicable to all pathological contexts. Despite this encouraging evidence, several limitations or issues still have to be overcome. Indeed, more than a few vaccines do not pass phases I of clinical trials either for toxicity issues and lack of immunogenicity in some individuals. What is missing? Part of the answer to this question could sit on human genetics and population variability. Syngeneic animal models are ideal settings in which the systems are pushed although they constitute a necessary and useful step preceding clinical trials.

Moreover, when translating vaccine testing from *in vivo* experiments on animals to *ex vivo* on human cells, often the opted choice are blood human cells, while in most of the cases vaccines will be administered in the skin, having a complete different DCs-based milieu (15). Crucially, Idoyaga and colleagues dissected the interindividual variability in skin-resident DCs, stressing the need of shedding light on the effects that genetics and environment imprint on DCs. It is compulsory to decode the complex scenario of human diversity to provide personalized therapies with increased efficacy. In the Omics era, systems biology and computational modeling integrate huge data-sets to address the urgent need of information on the global behavior. Indeed, Genome-wide association studies have provided insights into human genetics variants associated to the immunogenicity of vaccines (179, 180). Therefore, integration of “wet” evidence and “dry” notions may fasten the designing process and provide both efficient vaccine strategies and their predictive efficacy.

## Author Contributions

All authors listed have made a substantial, direct, and intellectual contribution to the work and approved it for publication.

## Conflict of Interest Statement

The authors declare that the research was conducted in the absence of any commercial or financial relationships that could be construed as a potential conflict of interest.
